# Evaluation of transitions from early hypertension to hypertensive chronic kidney disease, coronary artery disease, stroke and mortality: a Thai real-world data cohort

**DOI:** 10.3389/fcvm.2023.1170010

**Published:** 2023-05-02

**Authors:** Htun Teza, Suparee Boonmanunt, Nattawut Unwanatham, Kunlawat Thadanipon, Thosaphol Limpijankit, Oraluck Pattanaprateep, Anuchate Pattanateepapon, Gareth J. McKay, John Attia, Ammarin Thakkinstian

**Affiliations:** ^1^ Department of Clinical Epidemiology and Biostatistics, Faculty of Medicine Ramathibodi Hospital, Mahidol University, Bangkok, Thailand; ^2^ Division of Cardiology, Department of Medicine, Faculty of Medicine Ramathibodi Hospital, Mahidol University, Bangkok, Thailand; ^3^ Centre for Public Health, School of Medicine, Dentistry, and Biomedical Sciences, Queen’s University Belfast, Belfast, United Kingdom; ^4^ School of Medicine and Public Health, University of Newcastle, Newcastle, NSW, Australia

**Keywords:** cohort profile, hypertension, hypertension progression, multi-state model, real-world data, survival analysis, transition probability

## Abstract

**Objective:**

Systemic arterial hypertension (HT) is a major modifiable risk factor for cardiovascular disease (CVDs), associated with all-cause death (ACD). Understanding its progression from the early state to late complications should lead to more timely intensification of treatment. This study aimed to construct a real-world cohort profile of HT and to estimate transition probabilities from the uncomplicated state to any of these long-term complications; chronic kidney disease (CKD), coronary artery disease (CAD), stroke, and ACD.

**Methods:**

This real-world cohort study used routine clinical practice data for all adult patients diagnosed with HT in the Ramathibodi Hospital, Thailand from 2010 to 2022. A multi-state model was developed based on the following: state 1-uncomplicated HT, 2-CKD, 3-CAD, 4-stroke, and 5-ACD. Transition probabilities were estimated using Kaplan-Meier method.

**Results:**

A total of 144,149 patients were initially classified as having uncomplicated HT. The transition probabilities (95% CI) from the initial state to CKD, CAD, stroke, and ACD at 10-years were 19.6% (19.3%, 20.0%), 18.2% (17.9%, 18.6%), 7.4% (7.1%, 7.6%), and 1.7% (1.5%, 1.8%), respectively. Once in the intermediate-states of CKD, CAD, and stroke, 10-year transition probabilities to death were 7.5% (6.8%, 8.4%), 9.0% (8.2%, 9.9%), and 10.8% (9.3%, 12.5%).

**Conclusions:**

In this 13-year cohort, CKD was observed as the most common complication, followed by CAD and stroke. Among these, stroke carried the highest risk of ACD, followed by CAD and CKD. These findings provide improved understanding of disease progression to guide appropriate prevention measures. Further investigations of prognostic factors and treatment effectiveness are warranted.

## Introduction

Systemic arterial hypertension (HT) is a major modifiable risk factor of cardiovascular diseases (CVDs) and is associated with all-cause death (ACD) (1). This common non-communicable disease is characterized by persistent elevation of arterial blood pressure (BP), diagnosed as systolic blood pressure (SBP) ≥ 140 mmHg or diastolic blood pressure (DBP) ≥ 90 mmHg (2–4). About 1.28 billion adults worldwide are estimated to have HT, with almost half (46%) of them being unaware of the condition, hence the moniker “silent killer” as per the World Health Organization 2021 report (4). In Thailand, 25% of adults were diagnosed with HT according to the National Health Survey of 2014 (5), and HT is estimated to account for two-thirds of stroke and half of atherosclerotic coronary artery disease (CAD) events (6).

The etiology of HT involves the complex interplay of increased dietary salt intake, obesity, activation of neuro-hormonal systems such as the sympathetic nervous system and renin-angiotensin-aldosterone system (RAAS), as well as genetic predisposition (7), causing multi-system effects and long-term complications. Among these complications, chronic kidney disease (CKD) (8, 9), coronary artery disease (10, 11), stroke (12, 13) are frequent risks that cause premature death in HT patients (14–18). Progressive glomerulo-sclerosis is commonly observed in hypertensive CKD patients, with prevalence ranging from 60% to 90%, dependent on CKD stage and cause (19), and a previous study reported 30.9% incidence of CKD observed in hypertensive cohort (20). Furthermore, there is a bi-directional association, where sustained HT results in poorer kidney function, and progressive kidney function decline leads to worsening BP control. HT accelerates cholesterol-dependent atherogenesis and promotes atherosclerotic plaque development in the cerebrovascular circulation increasing the risk of ischemic stroke (21), and atherosclerotic CAD (22). Globally, CAD and stroke rank second and third among the causes of mortality and disability (23), and HT remains the most important risk factor of CAD and stroke (24–26).

HT and its complications have an impact on both individual-level quality of life and population-level economic health burden. A review estimated that the United States spent 193 billion USD in 2017 on HT management, not including economic loss caused by reduced productivity or increased premature death; direct cost (e.g., drugs, diagnostics, hospitalization, consultations) per person were estimated to be 6,250 USD (27). The financial burden is further increased when treating associated complications; for example, post-stroke hospitalization cost 15,415 USD per person in 2004 (28). Therefore, improved understanding of HT progression or transition to other conditions will lead to more appropriate, effective and timely treatment strategies.

A multi-state model of HT progression including CKD, CAD and stroke allows the estimation of time for disease progression between each disease state. Mortality associated with individual HT complications has been reported, e.g., stroke (*N* = 503) in Ethiopia (29), myocardial infarction (*N* = 2,336) (12) and CKD (*N* = 9,361) in the US (30), with several studies conducted in Asian populations using multi-state modelling. One study included 13,933 elderly Chinese subjects aged 60 years or older to assess prognostic factors and transitions from healthy state to cardio-metabolic disease and multi-comorbidity (respectively defined as one and two or more complications among HT, diabetes, CAD, and stroke), and death (31). However, this study also included non-hypertensive patients. A previous study in Italy reported association of non-communicable diseases including HT with adverse cardiovascular events and death (32). An Iranian study evaluated 3,002 hypertensive patients aged 50 years or older (33) to develop an illness-death model, known as a basic multi-state model, to estimate transition probabilities and prognostic factors for changing states from CVD-free HT to any CVD and ACD. However, this study considered both CAD and stroke as composite CVD events when, in reality, they have different prognoses. In addition, although CKD plays an important role in disease progression, this was not considered. Furthermore, HT disease progression in Southeast Asia may differ relative to other countries and ethnicities due to different prognostic factors, treatments, healthcare system, clinical practice guidelines, resource availability and accessibility, and reimbursement schemes.

As such, this large-scale, real-world dataset was constructed from a Thai teaching hospital. The study cohort sought to estimate the transition probabilities from HT without complication to any long-term complications including CKD, CAD, stroke, and ACD as well as transition probabilities between complications.

## Materials and methods

### Data design

This retrospective cohort study retrieved all routine patient electronic medical records from January 2010 to December 2022 from Ramathibodi Hospital, Bangkok, Thailand. Patients aged 18 years and over, diagnosed with HT, and followed up for more than one visit and longer than 30 days were identified and included. The study was approved by Ramathibodi Human Research Ethics Committee (COA.MURA 2021/512).

### Data sources and features

**Figure 1:**
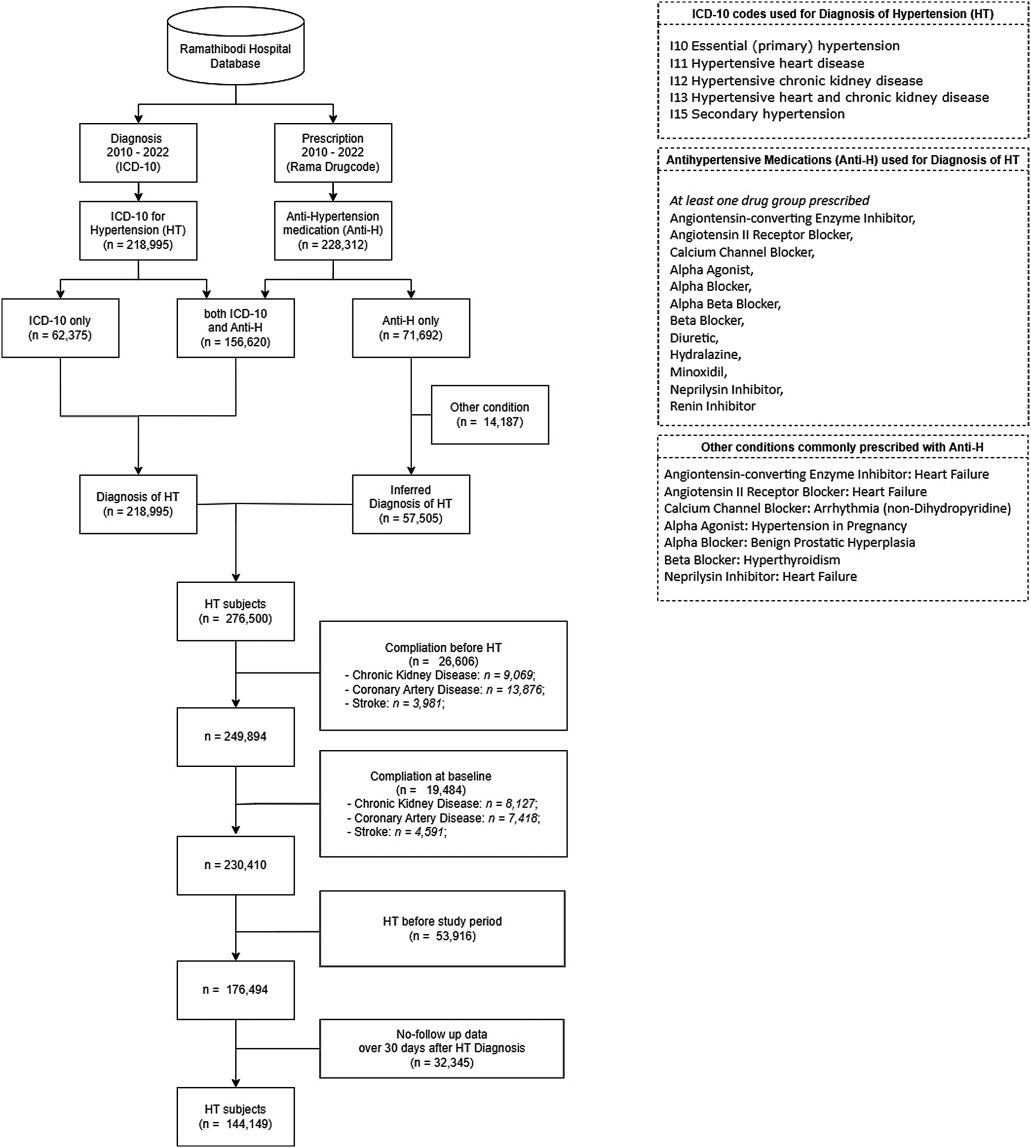
Data retrieval and processing flow.

Data from both routine clinical visits and hospital admission records were retrieved to assess the illness progression and identify conditions of interest based on the International classification of Diseases (ICD) Ninth (ICD-9) (34) and Tenth Revisions (ICD-10) (35). To develop the cohort, ICD-10 codes I10, I11, I12, I13 and I15 for HT were retrieved. To correct for potential under-coding (36), medication data were also retrieved to identify those taking alpha/beta blockers (BBs), calcium channel blockers (CCBs), agents acting on the renin-angiotensin system [i.e., angiotensin converting enzyme inhibitors (ACEIs), angiotensin II receptor blockers (ARBs)], and other HT related drugs (i.e., diuretics, minoxidil, and reserpine). However, beta blockers are also commonly prescribed to control other conditions such as hyperthyroidism. Therefore, patients who were prescribed a single anti-hypertensive medication along with other indications such as hyperthyroidism were excluded. We also used a 5 year “look back” period (2005–2010) to exclude those participants who were diagnosed or developed complications before study initiation. The data flow for identification of HT cohort is described in Figure 1.

Other covariates were extracted including: demographic data (i.e., age and sex), physical data (i.e., BP, heart and respiratory rates per minute, body temperature, and body mass index); laboratory data [i.e., lipid profiles (i.e., total cholesterol, high-density lipoprotein, low-density lipoprotein), kidney function [i.e., urine creatinine, estimated glomerular filtration rate (eGFR), urinary albumin-to-creatinine ratio], complete blood counts (i.e., hemoglobin, white blood cell count, red blood cell count) and troponin)] and prescribed medications (e.g., anti-hypertensive drugs, diabetic drugs, dyslipidemia drugs, drugs for atrial fibrillation, etc.).

Laboratory data were standardized to conventional units, while medications were converted to generic drugs and drug classes based on prescription and pharmacy dispensing information. Data across different information systems were linked and merged using de-identified hospital numbers and visit dates as shown in Supplement Figure 1. Given that patients were followed up at varying time intervals, data were aggregated to 180 day-intervals where possible.

### Complications of interest

Complications of interest associated with HT progression included CKD, CAD, stroke, and ACD. CKD was identified by ICD-10 and ICD-9 codes for renal replacement therapy. Also, eGFR was used to identify those who were not coded but had eGFR less than 60 ml/min/1.73 m^2^ on two consecutive occasions more than 90 days apart. The estimated GFR (ml/min/1.73 m^2^) was calculated based on the CKD epidemiology collaboration (CKD-EPI) equations (37). Similarly, CAD was identified using ICD-10 and ICD-9 codes as well as procedure codes for percutaneous coronary intervention (PCI), coronary artery bypass graft surgery (CABG), and troponin results higher than 14 ng/ml. Patients identified from troponin levels were verified using electrocardiogram findings within two weeks prior to or after the troponin test. Stroke was characterized as ischemic, hemorrhagic or transient ischemic attack, identified using ICD-10 codes. ACD and date were identified and retrieved from hospital databases. All ICD codes and criteria used to infer diagnoses are reported in Supplementary Table S1.

### Statistical analysis

**Figure 2:**
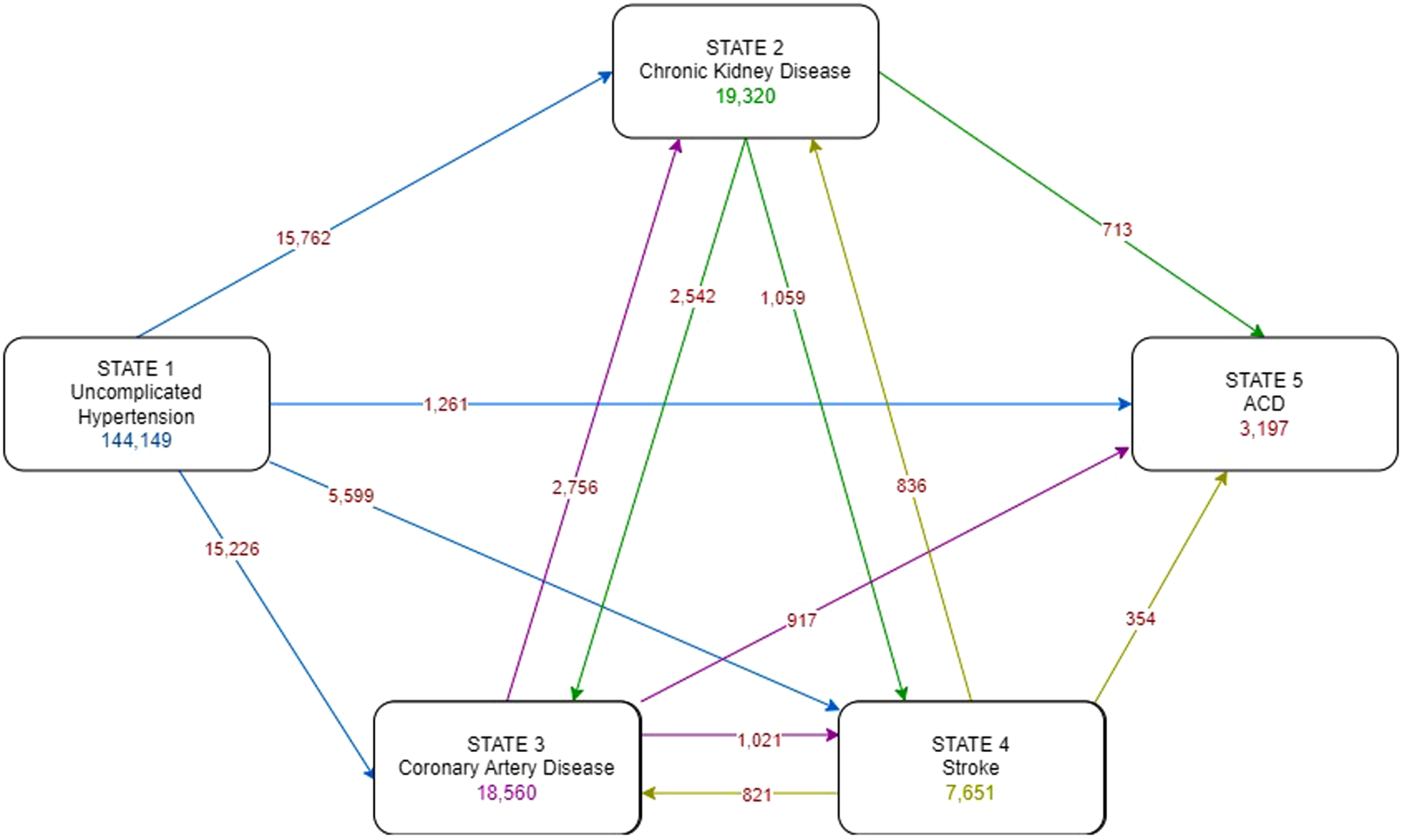
Multi-state diagram of progression of hypertension.

A multi-state model was developed, which consisted of three intermediate states (i.e., CKD, CAD, stroke) and the absorbing state of ACD, see Figure 2. Patients could directly move or transition from state 1 (complication-free) to state 2 (CKD), state 3 (CAD), state 4 (stroke), or even state 5 (ACD), whichever occurred first. Once they entered an intermediate state (2, 3, or 4), they could transition between them (i.e., 2 → 3, 2 → 4, 3 → 2, 3 → 4, 4 → 2, 4 → 3), then to ACD (e.g., 2 → 3 → 5, 2 → 4 → 5, 3 → 4 → 5, etc.), or directly to ACD (i.e., 2 → 5, 3 → 5, 4 → 5). Time from the initial state (date at HT diagnosis) to each intermediate or absorbing state (date at diagnosis of CKD, CAD, stroke, ACD), whichever occurred first, was calculated if such a transition was observed. Time from entry to the intermediate state to another state or absorbing state was also calculated if patients had subsequent transitions. In addition, patients were censored: (a) at 31st December 2022 if they were still complication free, (b) at the date of last encounter if lost to follow up.

The transition probabilities for 13 transition pathways from initial state to each intermediate and absorbing states (i.e., CKD, CAD, Stroke, and ACD), transitions between intermediate states, and intermediate states to ACD were estimated using a Kaplan–Meier test (38). Simultaneous transition to multiple complication states were considered accordingly, such as development of two complications recorded at the same visit was included in two analyses for both transition pathways. Transition probabilities for hazard functions were estimated using the lifelines package (39) (version 0.27.0) and Python (version 3.9.7) (40) within a Spyder integrated development environment (version 5.1.5) (41).

## Results

### Baseline characteristics

A total of 276,500 HT patients aged 18 years or over were identified using the ICD-10 codes and/or antihypertensive medications. Of these, 132,351 were excluded due to: (a) development of a complication before HT diagnosis (26,606 subjects: 13,876 patients with CAD, 9,069 with CKD and 3,981 with stroke with one patient that may have more than one complication at the same time), (b) complication diagnosed together with HT (19,484 subjects: 8,127 patients with CKD, 7,418 with CAD and 4,591 with stroke), (c) HT diagnosis preceding the study period (53,916 subjects), or (d) no follow up after 30 days from HT diagnosis (32,345 subjects). The remaining 144,149 patients were included in the study cohort, see Figure 1.

Baseline characteristics for study participants are presented in Table 1. The mean age was 57.8 ± 15.0 years with 60.6% being female. Average BMI was 25.9 ± 5.4 kg/m^2^ and average eGFR was 85.9 ± 25.4 ml/min/1.73m^2^. CCBs were the most commonly prescribed antihypertensive medication (42.8%). In addition, the median follow-up time was 3.6 years (range: 0.08–13.00) and the median number of visits was 33 (IQR: 11–76).

**Table 1:** Baseline characteristics of the hypertensive participants.

Characteristics	*N* = 144,149
**Demographics**	
Age, years, mean (SD)	57.8 (15.0)
**Sex, *n* (%)**	
Female	87,415 (60.6)
Male	56,734 (39.4)
BMI, kg/m^2^, mean (SD)	25.9 (5.4)
**Comorbidity**	
Diabetes Mellitus, *n* (%)	23,899 (16.6)
Dyslipidemia, *n* (%)	29,192 (20.3)
Atrial fibrillation, *n* (%)	2,093 (1.5)
**Laboratories**	
Total cholesterol, mg/dl, mean (SD)	2,196.5 (45.6)
HDL Cholesterol, mg/dl, mean (SD)	50.8 (13.8)
LDL Cholesterol, mg/dl, mean (SD)	122.2 (36.1)
Urine Creatinine, mg/dl, median (IQR)	0.3 (0.2, 12.3)
eGFR, ml/min/1.73 m^2^, mean (SD)	85.9 (23.4)
Troponin, ng/dl, median (IQR)	19.2 (6.7, 19.2)
**Medications used at baseline**	
Alpha/beta blocking agents, *n* (%)	28,366 (19.7)
CCBs, *n* (%)	34,395 (23.9)
ACEIs/ARBs, *n* (%)	24,536 (17.0)
Other hypertensive drugs, *n* (%)	9,163 (6.4)
**Medication used in cohort (i.e., prescribed at least once)**	
Alpha/beta blocking agents, *n* (%)	53,681 (37.2)
CCBs, *n* (%)	61,666 (42.8)
ACEIs/ARBs, *n* (%)	53,074 (36.8)
Other hypertensive drugs, *n* (%)	23,310 (16.2)

ACEIs, angiotensin converting enzyme inhibitors; BMI, body mass index; CCBs, calcium channel blockers; eGFR, estimated glomerular filtration rate; HDL, high-density lipoprotein; IQR, interquartile range; LDL, low-density lipoprotein; n/N, number of subjects; SD, standard deviation.

### Transition probabilities

**Figure 3:**
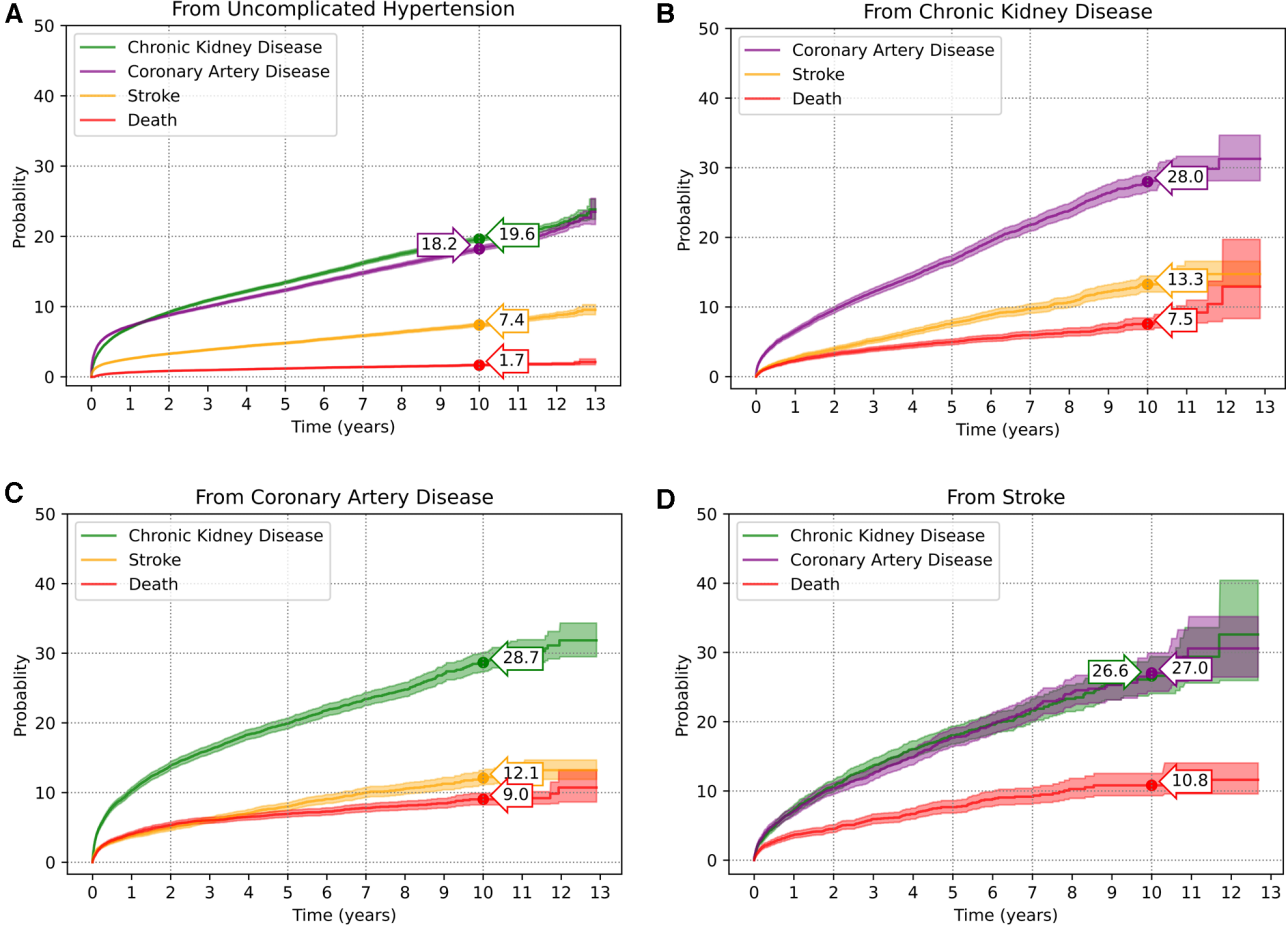
Transition probabilities of complication occurrences in hypertensive patients: (**A**) from uncomplicated HT to complications; (**B**) from chronic kidney disease to other complications; (**C**) from coronary artery disease to other complications; (**D**) from stroke to other complications.

As per the multi-state diagram in Figure 2, a total of 144,149 patients were included. Of these, 15,762 (10.9%), 15,226 (10.6%), 5,599 (3.9%) and 1,261 (0.9%) patients moved from the initial uncomplicated state directly to state 2-CKD, state 3-CAD, state 4-stroke, and state 5-ACD, respectively; the remaining 107,308 patients remained complication-free by the end of the study period, see Supplementary Table S2. Upon moving to an intermediate state (i.e., state 2-CKD, state 3-CAD, state 4-stroke), some patients further moved between other complication states, or ACD. As a result, 19,320 (13.4%), 18,560 (12.9%), and 7,651 (5.3%) patients were at subsequent risk of state 2-CKD, state 3-CAD, and state 4-stroke, see Figure 2. The transition probabilities for each state are presented in Figure 3. The transition probabilities (95% CI) for moving from an uncomplicated HT state to CKD, CAD, stroke, and ACD at 10 years were 19.6% (19.3%–20.0%), 18.2% (17.9%–18.6%), 7.4% (7.1%–7.6%), and 1.7% (1.5%–1.8%), respectively (Figure 3A).

Upon reaching the CKD state, HT patients had 10-year transition probabilities (95% CI) of 28.0% (26.6%–29.4%), 13.3% (12.1%–14.5%), and 7.5% (6.8%–8.4%) to progress to CAD, stroke, and ACD, respectively (Figure 3B). HT patients with CAD progressed to CKD, stroke, and ACD with 10-year transition probabilities of 28.7% (27.3%–30.1%), 12.1% (11.1%–13.2%), and 9.0% (8.2%–9.9%), respectively (Figure 3C). HT patients with stroke progressed to CKD, CAD, and ACD with 10-year transition probabilities (95% CI) of 26.6% (24.1%–29.4%), 27.0% (24.4%–29.9%), and 10.8% (9.3%–12.5%), respectively (Figure 3D). Further details on the transition probabilities of progressing to each state at 2-, 5-, 7- and 10-years are shown in Supplementary Tables S3–S6.

## Discussion

This study followed a retrospective cohort of 144,149 HT patients and their subsequent development of long-term complications from Ramathibodi Hospital, Bangkok, Thailand from 2010 to 2022. This large real-world Asian cohort allowed the estimation of the transition probabilities in individuals who were initially complication-free to progressive complication states and ACD. We found that HT patients were most likely to transition to CKD followed by CAD and then stroke with associated 10-year transition probabilities of 19.6%, 18.2%, and 7.4%, respectively. Among these complications, stroke had the highest risk of mortality followed by CAD in contrast to CKD, which had the lowest risk of death.

In addition to HT and traditional atherosclerotic risk factors, CKD also has been associated with accelerated atherosclerosis (42, 43). In our study, hypertensive patients with CKD had 10-year transition probabilities of 28.0% and 13.3% to CAD and stroke, respectively, which is similar to that reported previously, where HT patients with CKD had greater risk of developing CAD (44, 45) and stroke (46, 47) compared to uncomplicated HT patients. Conversely, patients with CAD and stroke were most likely to transition to CKD, with 10-year transition probabilities of 28.7% and 26.6% respectively, suggesting a bi-directional relationship (48, 49).

Once any complication had developed, HT patients were more likely to move to the absorbing state of ACD, with 10-year probabilities of 10.8%, 9.0% and 7.5% from each of the stroke, CAD, and CKD states, respectively. Although previous studies reported an elevated risk of mortality of 24% to 70% with decreased kidney function (50), our finding showed much more modest risk, which may be due to the Universal Healthcare Coverage in Thailand which has been available since 2002, including peritoneal dialysis since 2008 (51, 52). Subsidizing care improves treatment accessibility, reducing the mortality rate (53–55).

As shown in Figure 3, the transition from CKD to CAD increased at a higher rate over time compared to other competing states, with a sharp increase in the first year. Previous studies have reported that kidney disease and decreased renal function are risk factors for CAD (43, 56). American Heart Association Councils on Kidney recommend the patients with CKD be considered in the highest risk group for subsequent cardiovascular event (57). Conversely, transition from CAD to CKD was also observed to be the highest at 28.7% (27.3%–30.1%) for 10-years transition probability, compared to stroke and uncomplicated HT at 26.6% (24.1%–29.4%) and 19.6% (19.3%–20.0%) respectively.

The two-year transition probability for ACD was the highest for patients with CAD at 5.3% (5.0%–5.7%), with stroke as the leading cause of ACD for the remaining study period followed by CAD, i.e., 7.7% (6.8%–8.6%) vs. 6.9% (6.5%–7.4%) at 5 years, 9.2% (8.1%–10.4%) vs. 7.8% (7.2%–8.4%) at 7 years, and 10.8% (9.3%–12.5%) vs. 9.0% (8.2%–9.9%) at 10 years. While some previous studies reported conflicting findings (58, 59), others were similar to ours (60, 61). This likely reflects differing levels of cardiac care and access to revascularization procedures and should be further investigated.

To prevent late complications, BP control through lifestyle changes, including low-salt intake, and increased physical activity, effectively lowers BP and prevents target organ damage and its CVD sequelae (62, 63). Pharmacological therapy is very effective in lowering BP and in preventing CVD outcomes in most patients (64). Awareness of early complication such as declining of renal function is the early surrogate of CVD prevention, especially stroke and CAD which are the major cause of premature death in hypertensive patients (65, 66). Microalbuminuria has been shown to be an early marker of hypertensive renal disease (67). Pharmacologic therapy to reduce microalbuminuria is associated with delayed progression of renal disease (68). Furthermore, statin therapy should be beneficial for HT patients with CKD due to their demonstrated safety and efficacy for both lowering lipid levels and preventing CVD events in pre-end stage CKD (69, 70).

Our findings offer insight into HT progression in a Southeast Asian population. This large real-world cohort of HT patients allowed the estimation of transition probabilities of multiple complications simultaneously (13 transition pathways overall). Further prognostic factors should be considered to aid clinicians and patients delay disease progression, particularly in high-risk patients. In addition, the effectiveness of different HT treatments, cost-effectiveness or utility analysis should be undertaken for improved resource planning and utilization, especially in limited settings.

Some limitations could not be avoided. Our results are based on routine clinical practice data, thus, follow-up time intervals varied across individual patients, and lab test data and prescriptions also varied, leading to the aggregation of data in 180-day windows. Date of diagnosis for each state may be inaccurate if patients were diagnosed elsewhere prior to attending Ramathibodi Hospital. An inter-hospital data linkage should be sought to minimize this limitation. Prognostic factors for moving to complication-states are not considered and beyond the scope of this current study. Further studies should be conducted to simultaneously consider important prognostic factors such as age, body mass index, anti-hypertensive treatments, comorbidities (e.g., diabetes, dyslipidemia, etc.) in a multi-state model.

## Conclusions

A well-characterized cohort of 144,149 HT patients was constructed based on 13-year real-world clinical data. CKD was observed to be the most common complication in hypertensive patients, followed by CAD and then stroke according to 10-year risks. Among complication states, stroke had the highest risk of mortality followed by CAD, while CKD had the lowest. Early detection of declining renal function and tight BP control has been shown to reduce late complications. Tailoring antihypertensive medication to the clinical setting to achieve a lower BP goal is critical. This finding might help physicians better understand disease progression and guide appropriate and timely prevention measures. Further prognostic factors, treatment effectiveness/safety, and economic evaluation are warranted.

## Data Availability

The data analyzed in this study is subject to the following licenses/restrictions: The data can be shared on request to Department of Clinical Epidemiology and Biostatistics, Faculty of Medicine Ramathibodi Hospital, Mahidol University. Requests to access these datasets should be directed to https://www.rama.mahidol.ac.th/ceb/CEBdatawarehouse/Submittheproposal.
