# Supplementary material for: Evaluation of transitions from early hypertension to hypertensive chronic kidney disease, coronary artery disease, stroke and mortality: a Thai real-world data cohort
Source: Front Cardiovasc Med. 2023 May 2;10:1170010. doi: 10.3389/fcvm.2023.1170010 (PMC10191197; doi:10.3389/fcvm.2023.1170010)

Supplement table 1. Criteria of disease classifications used for patient identification

| Condition | Criteria |
| --- | --- |
| Hypertension (HT) | - International Statistical Classification of Diseases and Related Health Problems (ICD) tenth revision (ICD-10) codes for Hypertension [I10, I11, I12, I13, I15] - Use of at least one anti-Hypertensive medications |
| Chronic Kidney Disease (CKD) | - ICD-10 diagnosis for CKD ['I770', 'N18', 'N19', 'T824', 'T825', 'T827', 'T828', 'T829', 'T85611', 'T85621', 'T85631', 'T85691', 'T8571', 'T861', 'Z49', 'Z490', 'Z4901', 'Z4902', 'Z492', 'Z940', 'Z992'] - and ICD-9 procedures for renal replacement therapy ['38.93', '38.95', '39.27', '39.42', '39.43', '39.53', '39.95', '54.93', '54.98', '55.6'] - eGFR measurements less than 60 ml/min/1.73m2 for 2 consecutive tests of more than 90 days interval |
| Stroke | - ICD-10 diagnosis for Ischemic stroke ['I63', 'I64'] - ICD-10 diagnosis for Hemorrhagic strokes ['I60', ‘I61’, ‘I62’, 'I69'] - ICD-10 diagnosis for Transient Ischemic Attack [‘G45’] |
| Coronary Artery Disease (CAD) | - ICD-10 diagnosis for Coronary Artery Disease [‘I20’, ‘I21', 'I22', 'I23', ‘I24’, ‘I25’] - ICD-9 procedures for percutaneous coronary intervention (PCI) or coronary artery bypass surgery (CABG) ['00.4, '00.66', '17.55', '36.0', '36.1', '36.31', '36.99‘] - Troponin value more than 14 ng/ml with abnormal ECG result 14 days before and after |
| All-cause Death (ACD) | - Ramathibodi Hospital Death Registry |

Supplement Table 2 (a). Number of patients who remained and moved to intermediate and absorption states

|  | To | | | | | |
| --- | --- | --- | --- | --- | --- | --- |
| From |  | HT | CKD | CAD | Stroke | ACD |
|  | HT | 107,308 | 15,762 | 15,226 | 5,599 | 1,261 |
|  | CKD | 0 | 15,118 | 2,542 | 1,059 | 713 |
|  | CAD | 0 | 2,756 | 14,014 | 1,021 | 917 |
|  | Stroke | 0 | 836 | 821 | 5,680 | 354 |

*Note: Patients may be observed with more than one complications in the same visit. Such patients are included in each complications observed together. Refer to Table 2(b) for separate counts. HT: uncomplicated HT; CKD: chronic kidney disease; CAD: coronary artery disease; ACD: all-cause death*

Supplement Table 2 (b). Number of patients who remained and moved to intermediate and absorption states

|  | Total | HT | CKD | CAD | Stroke | Death | CKD+CAD | CKD + Stroke | CKD + Death | CAD + Stroke | CAD + Death | Stroke + Death | CKD + CAD + Stroke | CKD + CAD + Death | CKD + Stroke + Death | CAD + Stroke + Death | CKD + CAD + Stroke + Death |
| --- | --- | --- | --- | --- | --- | --- | --- | --- | --- | --- | --- | --- | --- | --- | --- | --- | --- |
| HT | 144,149 | 107,308 | 15,113 | 14,630 | 5,145 | 1,001 | 329 | 160 | 111 | 188 | 37 | 73 | 15 | 21 | 12 | 5 | 1 |
| CKD | 19,320 |  | 15,118 | 2,461 | 984 | 647 |  |  |  | 44 | 35 | 29 |  |  |  | 2 |  |
| CAD | 18,560 |  | 2,650 | 14,014 | 956 | 797 |  | 23 | 78 |  |  | 37 |  |  | 5 |  |  |
| Stroke | 7,651 |  | 807 | 796 | 5,680 | 328 | 14 |  | 15 |  | 11 |  |  |  |  |  |  |
| Death | 3,197 |  |  |  |  |  |  |  |  |  |  |  |  |  |  |  |  |

*HT: uncomplicated HT; CKD: chronic kidney disease; CAD: coronary artery disease; ACD: all-cause death*

Supplement table 3. Transition probabilities of uncomplicated hypertension to other complications

| **Years (%)** | **CKD** | **CAD** | **Stroke** | **ACD** |
| --- | --- | --- | --- | --- |
| 2 | 9.2  (9.0, 9.4) | 8.8  (8.6, 8.9) | 3.3  (3.2, 3.4) | 0.8  (0.8, 0.9) |
| 5 | 13.4  (13.2, 13.7) | 12.3  (12.1, 12.5) | 4.8  (4.7, 4.9) | 1.2  (1.1, 1.3) |
| 7 | 16.2  (15.9, 16.5) | 14.8  (14.5, 15.0) | 5.9  (5.7, 6.0) | 1.4  (1.3, 1.5) |
| 10 | 19.6  (19.3, 20.0) | 18.2  (17.9, 18.6) | 7.4  (7.1, 7.6) | 1.7  (1.5, 1.8) |

Numbers in cell are percentage (95% CI); *HT: uncomplicated HT; CKD: chronic kidney disease; CAD: coronary artery disease; ACD: all-cause death*

Supplement table 4. Transition probabilities of hypertension with chronic kidney disease to other complications

| **Years (%)** | **CAD** | **Stroke** | **ACD** |
| --- | --- | --- | --- |
| 2 | 9.5  (9.1, 10.0) | 3.9  (3.6, 4.3) | 3.2  (3.0, 3.5) |
| 5 | 16.7  (16.0, 17.4) | 7.6  (7.1, 8.2) | 5.0  (4.6, 5.4) |
| 7 | 21.8  (20.9, 22.7) | 9.8  (9.1, 10.5) | 5.9  (5.5, 6.5) |
| 10 | 28.0  (26.6, 29.4) | 13.3  (12.1, 14.5) | 7.5  (6.8, 8.4) |

Numbers in cell are percentage (95% CI); *HT: uncomplicated HT; CKD: chronic kidney disease; CAD: coronary artery disease; ACD: all-cause death*

Supplement table 5. Transition probabilities of hypertension with coronary artery disease to other complications

| **Years (%)** | **CKD** | **Stroke** | **ACD** |
| --- | --- | --- | --- |
| 2 | 13.9  (13.3, 14.5) | 4.9  (4.5, 5.3) | 5.3  (5.0, 5.7) |
| 5 | 19.9  (29.2, 20.7) | 8.0  (7.5, 8.6) | 6.9  (6.5, 7.4) |
| 7 | 23.4  (22.5, 34.4) | 10.0  (9.3, 10.7) | 7.8  (7.2, 8.4) |
| 10 | 28.7  (27.3, 30.1) | 12.1  (11.1, 13.2) | 9.0  (8.2, 9.9) |

Numbers in cell are percentage (95% CI); *HT: uncomplicated HT; CKD: chronic kidney disease; CAD: coronary artery disease; ACD: all-cause death*

Supplement table 6. Transition probabilities of hypertension with stroke to other complications

| **Years (%)** | **CKD** | **CAD** | **ACD** |
| --- | --- | --- | --- |
| 2 | 10.7  (9.9, 11.6) | 10.3  (9.5, 11.2) | 4.5  (4.0, 5.1) |
| 5 | 18.0  (16.8, 19.3) | 17.7  (16.5, 19.0) | 7.7  (6.8, 8.6) |
| 7 | 21.6  (20.0, 23.3) | 21.9  (20.2, 23.6) | 9.2  (8.1, 10.4) |
| 10 | 26.6  (24.1, 29.4) | 27.0  (24.4, 29.9) | 10.8  (9.3, 12.5) |

Numbers in cell are percentage (95% CI); *HT: uncomplicated HT; CKD: chronic kidney disease; CAD: coronary artery disease; ACD: all-cause death*

Supplement Figure 1. Data extraction and linkage across different information systems


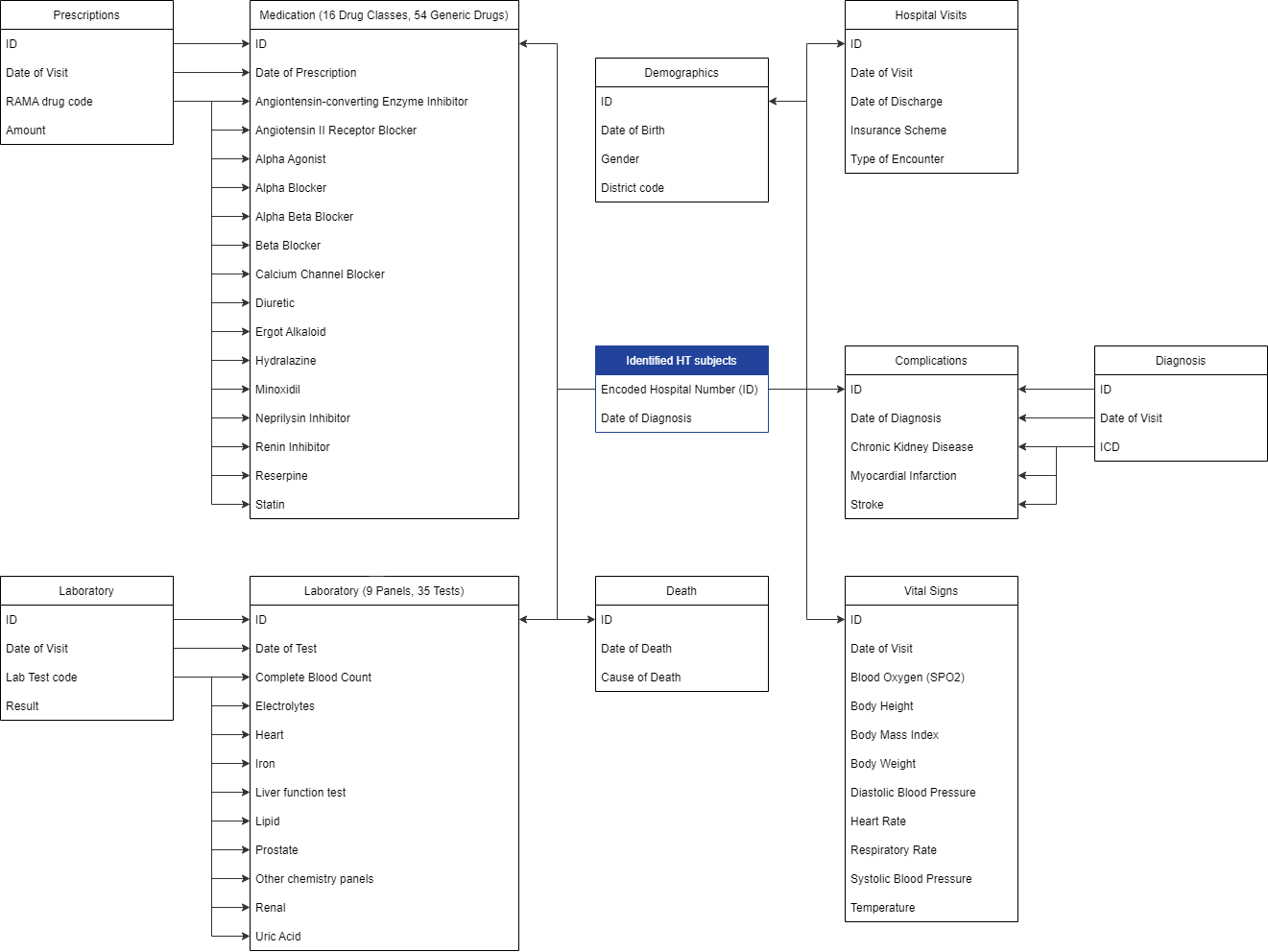

Supplement: Supplementary file 1 [file Datasheet1.docx]
